# Evaluation of the utilization of the preanaesthetic clinics in a University teaching hospital

**DOI:** 10.1186/1472-6963-6-59

**Published:** 2006-05-23

**Authors:** Seetharaman Hariharan, Deryk Chen, Lorna Merritt-Charles

**Affiliations:** 1 Anaesthesia and Intensive Care Unit, The University of the West Indies, Eric Williams Medical Sciences Complex, Trinidad, West Indies

## Abstract

**Background:**

Dedicated out-patient preanaesthetic clinics are relatively recent phenomenon and information is sparse from developing world. This study attempted to evaluate the utilization of adult and paediatric preanaesthetic clinics and its impact on the cancellations of surgery in Trinidad.

**Methods:**

All patients scheduled to have elective surgery during the period of twelve weeks were enrolled for prospective collection of data including demographics, the admitting diagnoses, surgical procedure, category of surgery and specialty, and the patients' attendance to preanaesthetic clinics. Cancellations on the day of surgery along with reasons were recorded. The difference between patients who attended and did not attend the clinic was analysed.

**Results:**

Of 424 patients scheduled for procedures during the study period, 213 were adults and 211 were children. Overall 39% of adults and 46% of the children scheduled for surgery had previously attended the preanaesthetic clinic. Among adults, general surgery patients were the largest majority to attend the preanaesthetic clinic. The paediatric preanaesthetic clinic was mostly utilized by paediatric general surgery. Overall 30% of procedures in adults and 26% of those in children were cancelled. There was a statistically significant difference in cancellations between patients who attended and did not attend the preanaesthetic clinic (p = 0.004). There was a 52% more chance of the procedure getting cancelled if the patient did not attend the clinic.

**Conclusion:**

The study highlights the inadequate use of the preanaesthetic clinics and the impact of the clinics on last-minute cancellations.

## Background

Preanaesthetic assessment of a surgical patient is done differently in various settings. In an outpatient setting this may be done by administering a questionnaire by the nursing or medical staff, or assessing the patient in a dedicated preanaesthetic out-patient clinic. In an in-patient setting the patient on the ward may be referred to the anaesthetist preoperatively for preoperative evaluation and optimization. Although in many institutions the anaesthetists get to see the patient on the day of the procedure in the patient-waiting room, there is evidence that a preanaesthetic assessment well before the procedure plays a vital role in avoiding last-minute cancellations and delays of surgical procedures [1]. There have been many published reports to show that these clinics have immensely helped to avoid inconvenience to the patients and staff alike [2,3].

Anaesthesia now being in the process of metamorphosis to "perioperative medicine", preanaesthetic clinic is being considered as one of the important domains of the anaesthetists [4]. These clinics greatly assist in optimizing patients in relation to the perioperative period. In addition to being cost-beneficial avoiding unnecessary investigations, this also offers the anaesthetist an opportunity of establishing a rapport with the patient, well in advance [5]. Dedicated anaesthetic clinic is a relatively recent phenomenon and data from the developing world is sparse. Since the inception of the preanaesthetic clinic in our hospital, the only one of its kind in the Caribbean, its utilization has not been studied. This study primarily analyzes the utilization of the adult and paediatric preanaesthetic clinics. Additionally it tests the hypothesis that whether the assessment of a surgical patient in the preanaesthetic clinics have any impact on the rate of "last-minute" cancellations of surgical procedures in both adult and paediatric patients.

## Methods

The study was conducted in Eric Williams Medical Sciences Complex, Trinidad, a tertiary care teaching hospital, affiliated to the University of West Indies. Trinidad and Tobago is a twin-island nation of the English-speaking Caribbean with a population of 1.3 million. Despite being one of the economically affluent countries in the Caribbean, it is still a developing country.

The hospital has two out-patient preanaesthetic clinics established a decade ago, one each for adult and paediatric patients and operate once a week. Patients are referred to these clinics from various surgical specialties the week before the scheduled surgical procedure. The patients are required to initially fill a self-administered questionnaire and a nurse records the vital signs. Resident doctors clinically assess every patient, under the supervision of a consultant anaesthetist. The assessment form is appended to the patient notes. The parent surgical unit is informed of any further referrals and/or investigations required, as well as the decision regarding the patient if the procedure needs to be postponed due to anaesthetic reasons.

Although the hospital has eight operating rooms, due to lack of resources only three to four operating rooms are functional. On a daily basis two operating rooms are used exclusively for ambulatory surgery and one for inpatients. The fourth operating room will be usually utilized by special programmes such as cardiac surgery, which is run as a quasi-private programme and hence was not included in the present study. The scheduled operating lists are sent from the Department of Surgery to the sister-in-charge of the operating rooms who manages the floor of the operating rooms. Due to consistent lack of resources there has been a problem in distributing the available operating time to the various specialties. There have been no structured scheduling procedures and back-up schedules in the event of cancellations.

Approval of the University Ethics Committee was obtained before the study and the requirement for informed consent from patients was waived because of the observational nature of the study. All public patients scheduled for elective surgical procedures consecutively over a period of twelve weeks from June 2002 through August 2002 were included for prospective collection of data. Private patients were excluded from the study because of administrative reasons. A pilot study was conducted over a week to familiarize the students collecting the data regarding the exact methodology and also to calculate the sample size.

For detailed evaluation, a sample size was determined to give adequate power to the findings of the study, based on figures for percentage cancellations derived from a pilot study. 30 percent of procedures were cancelled during the pilot study and this figure was used for the calculation of the sample size, which was derived at 322.

A data collection form was used to collect information regarding the demographic data such as the age and gender of the patients, the admitting diagnoses, surgical procedure scheduled, category of surgery and service offering the procedure. These data were recorded for all patients scheduled in the three operating theatres of the hospital. Because the hospital has separate preanaesthetic clinics for adults and children we collected and grouped the data with respect to these two age groups of patients.

All the case notes of the patients were analyzed to see if they had attended the preanaesthetic clinic anytime before the procedure and had been assessed by an anaesthetist in the clinic. Cancellations of the surgical procedures on the day of surgery were recorded in all the operating rooms. For every patient whose surgical procedure was cancelled on the day, the reasons for cancellation were noted in the data collection form, which were grouped into major categories namely patient's not showing up for surgery, unavailability of medical and/or nursing staff, inadequate patient preparation as decided by the consultant anaesthetist, acute patient illness, surgeon's decision to postpone, and miscellaneous reasons such as unavailability or failure of equipment, unavailability of linen, etc.

Data were entered into and analyzed using the SPSS version-12 (Chicago, IL, USA) software. Chi squared analysis and Mantel-Haenszel common odds ratio was calculated to analyze the impact of attending the preanaesthetic clinic on cancellations and the statistical significance was set at a 'p' value of less than 0.05.

## Results

During the twelve-week period of study, a total of 424 patients, scheduled for elective surgical procedures were enrolled into the study. Among these, 213 (50%) were adults and 211 (50%) were paediatric patients. Overall 181 patients (43%) attended the preanaesthetic clinic. 84 (39%) adults and 97 (46%) children attended the preanaesthetic clinic before the scheduled surgical procedure.

**Figure 1:**
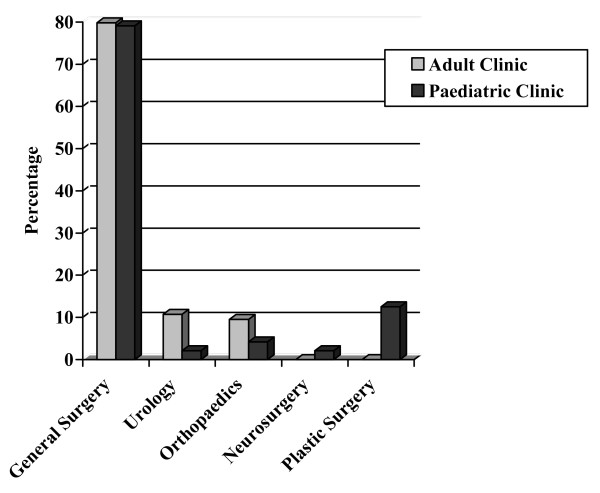
**Utilization of preanaesthetic clinics by surgical specialties**. (Percentage refers to the proportion of patients with respect to total attendance of the preanaesthetic clinics)

The utilization of the preanaesthetic clinic according to the different surgical specialties is shown in Figure 1. Among adults general surgical patients were the largest group to attend the preanaesthetic clinic (80%) followed by urology (11%) and orthopaedic surgery (10%). No patients from neurosurgery and plastic surgery attended the clinic. The paediatric preanaesthetic clinic was attended by paediatric surgery patients (79%), followed by orthopaedic surgery (13%), urology (4%) and neurosurgery and plastic surgery (2% each).

Overall there were 117 (27.6%) cancellations of the scheduled procedures. 63 out of 213 (30%) procedures in adults and 54 of 211 (26%) procedures in children were cancelled. The proportion of patients who had their procedures cancelled despite attending preanaesthetic clinic was 20% in adults and 21% in children. However the proportion of cancellations in patients who did not attend the preanaesthetic clinic was 36% in adults and 30% in children. Thus the overall incidence of cancellations was higher in patients who did not attend the preanaesthetic clinic, when compared to patients who attended the clinic. A Chi-square evaluation of the difference between the cancellations showed statistical significance (Pearson's Chi-square value: 8.09, df: 1, p = 0.004).

A Mantel-Haenszel common odds ratio estimate in relation to cancellation of procedure and attending preanaesthetic clinic was 0.52, (95% confidence intervals being 0.33, 0.82) (p = 0.005) implying that there was a 52% more chance of the procedure getting cancelled if the patient would not have attended preanaesthetic clinic.

The recorded reasons for cancellations among patients who attended and did not attend the preanaesthetic clinic are shown in Table 1. Patients' not "showing up" was the most common reason for cancellation. Among adults, general surgical patients were cancelled more frequently (64%) followed by urology (21%), orthopaedic surgery (13%) and neurosurgery and plastic surgery (2% each). In children, paediatric surgery bore the major brunt of cancellations (70%) followed by plastic surgery (17%), orthopaedic surgery (7%) and urology (6%). Neurosurgery had no cancellations during the study period.

**Figure 2:**
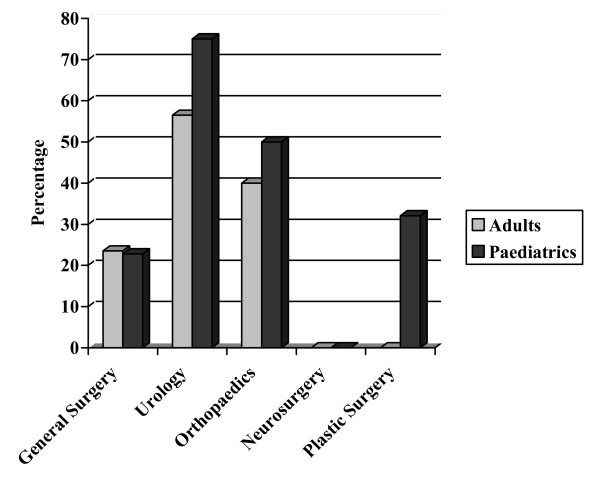
**Cancellation by surgical specialties**. (Percentage refers to the proportion of patients cancelled despite attending the preanaesthetic clinic)

Figure 2 shows the proportion of patients in each specialty who had their procedures cancelled despite attending the preanaesthetic clinics.

## Discussion

The present study shows that there has been an inadequate utilization of the preanaesthetic clinic by the surgical specialties associated with a very high rate of cancellation of 30%.

There are many possible reasons for the high cancellation rate. The 'miscellaneous' category of reasons for cancellation in the present study was a high proportion of cancellations (Table 1) and this consisted of reasons such as unavailability of equipment, breakdown of equipment, lack of linen due to either shortage of linen or breakdown of the central sterilization equipment, unavailability of anaesthetic technicians, unavailability of resources to clean the operating rooms from the day before, etc. This is a typical 'third world' phenomenon and because of the lack of coordination of different departments involved in the functioning of operating rooms and lack of efficient management of operating theatre floor, many surgical procedures were cancelled. Although a similar finding has been reported by earlier studies both from the developed world as well as from the Caribbean, the reported rate of cancellations was not as high as the present study [6,7]. Cancellations of this nature, despite patients attending the preanaesthetic clinics may dissuade the surgical specialties referring their patients to the clinics on a regular basis.

As mentioned earlier two of the three functioning operating rooms in our hospital are exclusively dedicated to ambulatory surgery. Many of these patients belong to physical status of American Society of Anesthesiologists (ASA) physical status I and II, and many surgeons do not feel the need of referring such patients to the preanaesthetic clinics. Since the clinics operate only once a week, many surgical specialties having out-patient clinics on different days find it difficult to ask the patient to return on a different day for preanaesthetic evaluation.

Additionally because our hospital predominantly caters service to paediatric patients, there were cancellations due to sudden unexpected upper respiratory symptoms in children on the day of surgery. Cancellation due to this reason has been well documented and may not be influenced upon by evaluation in the preanaesthetic clinic [8].

There have been many published reports highlighting the importance of preanaesthetic clinics and their beneficial effects [9-11]. Most importantly the clinics play a part in evaluating surgical patients with co-morbid illnesses and assist in preoperative optimization. Improper preoperative preparation has been reported as one of the major reasons for cancellations of surgical procedures [12]. This issue could be addressed by implementing early warning parameters, and in complicated cases this could be easily done in an outpatient environment such as the anaesthetic clinic [13]. Disagreements between the surgeons and anaesthetists in many issues such as the amount of banked blood available for a particular procedure may well be addressed by a preanaesthetic evaluation in the clinic. In the present study all patients whose procedures were cancelled because of improper preoperative preparation belonged to the group who did not attend the preanaesthetic clinic (Table 1).

Despite clear advantages, there have been controversies regarding the establishment and utility of these clinics [14]. The major dissident view is that in many settings it is impossible to spare anaesthetic staff exclusively for these clinics due to shortage of both material and human resources [15]. In a developing country such as ours, establishing exclusive anaesthesia clinics may be defied by cost factor. However, many studies have shown that these clinics are cost effective not only with respect to avoiding unnecessary investigations but also for the hospital administration in allocating resources for such a clinic [4,16,17]. In our situation, although the hospital has staff shortages, this is the only hospital in the Caribbean to establish and run two clinics – one each for adult and paediatric, without interruption for the past ten years.

Another controversy is that the surgical patient may be assessed by a different anaesthetist who may not actually conduct the anaesthesia for the given patient [18]. Although this may be true, there can be no doubt that complicated cases who require detailed evaluation need not wait until the day before surgery for evaluation and any anaesthetist in a particular setting could evaluate and offer advise in general. The specific anaesthetic techniques may vary but the common perioperative implications may be easily addressed. In our situation, we do respect the views of a colleague consultant anaesthetist regarding a patient's general assessment and the so-called 'fitness' for anaesthesia.

There is a suggestion that patients should undergo a preanaesthetic evaluation by questionnaire administration which would be scrutinized by staff in the clinic who will then decide if the patient needs to be further evaluated by an anaesthetist [19]. A nurse led questionnaire evaluation has shown to impact on the cancellation rate of surgical procedure [20]. Presently, our preanaesthetic clinic requires the patient to fill an initial questionnaire administered which form and all patients are assessed by residents who seek the consultant's opinion for major cases. We suggest that a future prospective study in our setting to triage patients and compare the effect of questionnaire evaluation alone and complete evaluation in the clinic may throw some more light on the impact of these clinics on cancellation rates.

There were some limitations to the present study. We could not clearly establish the beneficial impact of the clinic probably due to the duration of the study. Although we had a reasonable sample size, if the study would have been continued for a longer period, the advantages of the preanaesthetic clinic could have been found better.

## Conclusion

The study highlights the inadequate use of the preanaesthetic clinics and the impact of the clinics on last-minute cancellations.

## Competing interests

The author(s) declare that they have no competing interests.

## Authors' contributions

SH coordinated the study, interpreted the data, statistically analysed the data and drafted the manuscript. DC conceived of the study, and participated in its design and coordination and revised the manuscript. LMC participated in the design of the study and revised the manuscript. All authors read and approved the final manuscript.

## Pre-publication history

The pre-publication history for this paper can be accessed here:



## Figures and Tables

**Table 1:** Reasons for cancellations.

**Reasons**	**Patients who attended PAC(n = 37) (%)**	**Patients who did not attend PAC(n = 80) (%)**
'No show' of patients	20 (54)	23 (29)
Unavailability of staff	4 (11)	7 (9)
Insufficient operating time	11 (30)	1 (1)
Improper preoperative preparation	0	6 (8)
Acute patient illness	0	8 (10)
Surgeon's decision to postpone	0	2 (2)
Miscellaneous*	2 (5)	33 (41)

*Reasons such as unavailability of linen and equipment, failure of equipment, etc.
